# Climate change, cultural continuity and ecological grief: Insights from the Sámi Homeland

**DOI:** 10.1007/s13280-024-02012-9

**Published:** 2024-04-13

**Authors:** Inkeri Markkula, Minna Turunen, Taru Rikkonen, Sirpa Rasmus, Veina Koski, Jeffrey M. Welker

**Affiliations:** 1 Ecology and Genetics Research Unit, University of Oulu, Oulun Yliopisto, PL 8000, 90014 Oulu, Finland; 2 Arctic Centre, University of Lapland, Pohjoisranta 4, 961010 Rovaniemi, Finland; 3 Natural Resource Institute Rovaniemi, Ounasjoentie 6, 96200 Rovaniemi, Finland; 4 Department of Biological Sciences, University of Alaska Anchorage, 211 Providence Drive, CPSB 101, Anchorage, Alaska 99508 USA; 5 University of the Arctic (UArctic), Rovaniemi, Finland

**Keywords:** Atlantic salmon, Climate change, Ecological grief, Environmental change, Reindeer herding, Sámi Homeland

## Abstract

Arctic regions are warming significantly faster than other parts of the globe, leading to changes in snow, ice and weather conditions, ecosystems and local cultures. These changes have brought worry and concern and triggered feelings of loss among Arctic Indigenous Peoples and local communities. Recently, research has started to address emotional and social dimensions of climate change, framed through the concept of ecological grief. In this study, we examine sociocultural impacts of climate change and expressions of ecological grief among members of reindeer herding communities in the Sámi Homeland in Finland. Results indicate that ecological grief is felt in connection to major environmental concerns in the area: changes in winter weather and extreme weather events, Atlantic salmon decline and land use changes, which all have cultural and social consequences. Our results indicate that ecological grief is strongly associated with ecological losses, but also with political decisions regarding natural resource governance.

## Introduction

Arctic regions are warming four times faster than the global average (Rantanen et al. 2022), and warming has already caused significant changes in local weather, snow and ice conditions (Bailey et al. 2021), ecosystems and species communities (AMAP 2017; Buchwal et al. 2020). Alterations in climate and ecosystems are increasingly affecting the lives of Arctic Indigenous Peoples and local communities, whose livelihoods and cultures depend on seasonal cycles of fisheries and wildlife, predictable weather patterns and ecosystem health (Cunsolo et al. 2013, 2015; Jaakkola et al. 2018; Richert et al. 2021). A growing and shared concern is felt among Arctic peoples that the world they once knew no longer exists, and global climate change is increasingly embedded within their everyday experiences (Cunsolo and Ellis 2018; Watt-Cloutier 2018).

Previous studies have documented how climate change can cause disruptions to place attachment among Indigenous Peoples and local communities who live in close relationship with the surrounding nature, as well as loss of cultural identity and traditional ways of knowing when traditional knowledge becomes outdated (Cunsolo et al. 2012, 2013, 2015). Alterations in weather patterns, snow and ice conditions, degradation of ecosystems and regional decline in native plant and animal species have been associated with a decrease in well-being and an increase in stress and despair in particular among Indigenous Peoples’ communities (e.g., Jaakkola et al. 2018; Cunsolo et al. 2020). A recent study conducted in the Nunatsiavut region in Canada found significant relations between warmer temperatures (i.e., above −5 °C) and increased incidence rate of daily mental health-related clinic visits among Inuit population (Middleton et al. 2021).

New concepts related to peoples’ lived experiences of climate change have emerged in research and public discourse, including climate change worry, eco-anxiety, solastalgia, climate grief and ecological grief (Ojala et al. 2021). According to Cunsolo and Ellis (2018), ecological grief is a natural response to ecological degradation and loss, especially among people whose lives and cultures are entwined with the natural environment. Ecological grief has been defined as “the grief felt in relation to experienced or anticipated ecological losses, including the loss of species, ecosystems and meaningful landscapes due to acute or chronic environmental change” (Cunsolo and Ellis 2018). The concept of ecological grief can be used to reveal personal and collective responses to ecological loss, to define what climate-related losses matter to local people, and to identify ways of coping with emotions arising from climate change and ecological loss (Cunsolo and Ellis 2018; Ojala et al. 2021).

Previous research has documented ecological grief in relation to climate change among Indigenous Peoples and farmers in different parts of the world (e.g., Cunsolo 2012; Cunsolo et al. 2012; Ellis and Albrecht 2017; Amoak et al. 2023). In the Arctic region, ecological grief in relation to warming temperatures and sea ice degradation (Cunsolo 2012; Cunsolo et al. 2012, 2013, 2015; Durkalec et al. 2015), forest fires (Dodd et al. 2018) and decline in caribou populations (Cunsolo et al. 2020) has been reported from Northern parts of Canada and Greenland. To date, the concept has not been applied to research conducted in other parts of the Arctic. Moreover, grief experienced in response to ecological losses is often left unconsidered in climate change narratives, policy and research (Cunsolo and Ellis 2018). According to Cunsolo and Landman (2017), thinking with and through ecological grief has potential to change ethical and political discussions of climate change. Grieving is also a common practice shared across cultures, and addressing ecological grief can shed light on cultural, spiritual and intangible values and meanings, which often receive little attention in studies on climate change impacts, as well as in land-use planning and decision-making processes (see, e.g., Reyes-Garcia et al. 2019; Howard 2020).

This study focuses on expressions of ecological grief among reindeer herders in the Sámi Homeland in Finland, where climate change and land use conflicts pose major—and growing—challenge for the continuity of traditional livelihoods and Sámi culture (Jaakkola et al. 2018). Warming has been rapid in the area over the past three decades, particularly in winter, and recent years have seen repeated extreme winter weather events, including, e.g., exceptional snow depths and repeated freeze-thaw cycles, alterations in ecosystems, decline in culturally important species and invasion of alien fauna in the Sámi Homeland region (Luomaranta et al. 2019; Markkula et al. 2019; Kumpula et al. 2020, 2022).

The Sámi Homeland is a legally defined, traditional home region of the Sámi. The Constitution of Finland (731/1999) secures the Sámi people’s right to maintain and develop their own language and culture, including reindeer herding and other traditional livelihoods such as fishing, hunting, gathering and handicrafts. Discussion on Indigenous Sámi rights to land and waters has been going on for a long time in Finland (Joona 2020). Most of the land in the Sámi Homeland continues to be state-owned, and the traditional livelihoods need to compete with other land uses, including forestry, tourism, mining and wind power, making their adaptation to climate change even more challenging (e.g., Landauer et al. 2021).

In this study, we first address observed environmental changes and their sociocultural impacts, more precisely, impacts on cultural continuity and well-being, in the Sámi Homeland. Second, we examine how ecological grief is expressed by reindeer herders and members of Sámi community, whose lives and livelihoods are closely connected to a rapidly changing environment. Our study is based on interviews and a public participatory geographic information system (PPGIS) survey conducted in two municipalities in Northern Finland.

## Materials and methods

### Research area

**Fig. 1:**
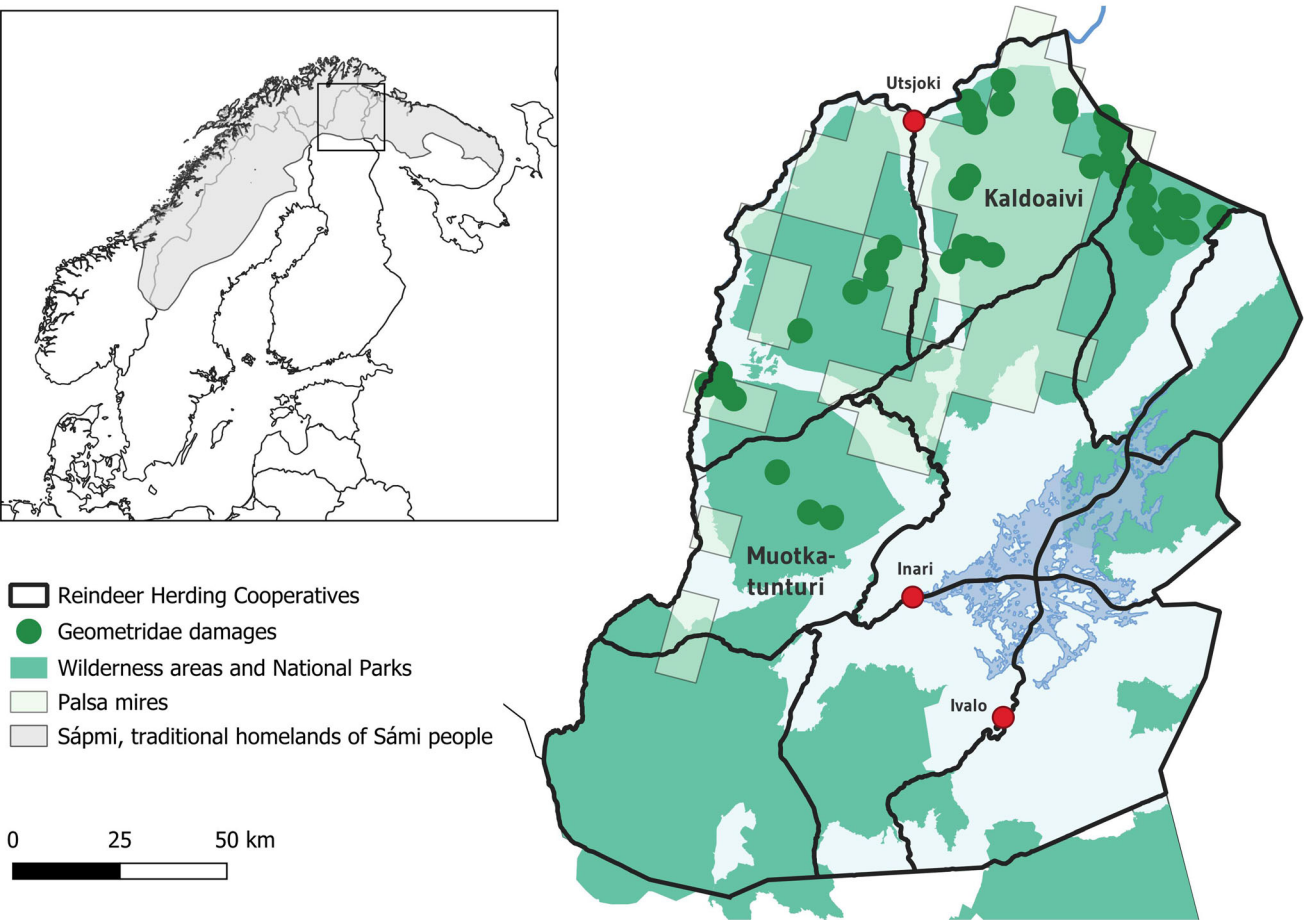
Research area. Map: Arto Vitikka, Arctic Centre, University of Lapland. Data sources: Arctic Indigenous languages and revitalization: an online educational resource (2023). Language speaker areas map and dataset (https://arctic-indigenous-languages-uito.hub.arcgis.com/), Palsa mires, Wilderness areas, SYKE: Luontotyyppien levinneisyyden raportointiaineisto (2019). Geometridae damages, Ylä-Lapin kaukokartoitushanke (2023)

This study was conducted in two municipalities in Northern Finland: Inari (Aanaar in Sámi language) and Utsjoki (Ohcejohka). The study area belongs to the subarctic region and is situated in the specific reindeer herding area and within the Sámi Homeland (Fig. 1). The reindeer herding area in Finland currently consists of 54 reindeer herding cooperatives, ten of which are situated in the study area: Kaldoaivi, Paistunturi Muotkatunturi, Hammastunturi, Ivalo, Näätämö, Vätsäri, Muddusjärvi, Sallivaara and Paatsjoki.

### Environmental concerns in the area

#### Climate change

Impacts of climate change are experienced in the study area particularly in winter. The length and characteristics of seasons, the duration of snow cover, and the amount and structure of snow have all changed over the last decades, the average temperature has risen by 2.3 °C since the post-industrial period, and the snow-free season is becoming longer (Luomaranta et al. 2019; Rasmus et al. 2020). In recent years, reindeer herders in the study area have needed to cope with extremely difficult snow conditions. Exceptional snow depths during winter 2019–2020 led to 15 000 reindeer deaths, and during winter 2021–2022 pasture conditions were unusually difficult in some parts of the study area due to basal ice and mold formation (Kumpula et al. 2020, 2022).

One significant environmental change that the area has experienced is the decline of Atlantic salmon (*Salmo salar*) populations in the River Teno (Deatnu) over the last decade. Reasons behind the decline include rising sea water temperatures, changes in salmon prey distributions, overfishing and fish farming in the Barents Sea (e.g., Czorlich et al 2022; Dadswell et al 2022). The River Teno, which runs through the Utsjoki municipality, along the border of Finland and Norway, is one of the most significant spawning rivers for Atlantic salmon in Europe (Hiedanpää et al. 2020). Fishing has a long tradition in the area, and Atlantic salmon fishing is a foundational part of Sámi culture. Many reindeer herders practice fishing during the summer months as an economic or subsistence activity, and Atlantic salmon has a high economic importance for the Utsjoki municipality (Knuuttila et al. 2020). In 2017, the governments of Finland and Norway ratified a fishing agreement (Teno Fishing Act 2017) in order to halt the decline of Atlantic salmon by reducing fishing volumes by 30%. The regulations were received with concern and anger among the Sámi population, who found them unfair and harmful for the Sámi fishing culture (Hiedanpää et al. 2020), feeling that their views are not adequately considered and that their traditional knowledge was not valued in resource governance of the river (Holmberg 2018). However, the Teno Fishing Act did not halt the decline in Atlantic salmon populations, and in 2021, Finland and Norway agreed on a temporary fishing ban for Atlantic salmon on the River Teno. The decision was opposed by the Sámi parliament of Finland. In their statement, the Sámi parliament noted that a complete fishing ban violated the rights of the Sámi under the Constitution of Finland to maintain and develop their culture (Finnish Constitution §17, 1999), and suggested that the restrictive measures should primarily be directed at sport salmon fishing rather than at traditional fishing (Sámi parliament 2021). In summer of the same year, a dramatic increase in pink salmon (*Oncorhynchus gorbuscha*)—an invasive species—took place in the River Teno. By summer 2023, the pink salmon population had reached 100 000, outnumbering the native Atlantic salmon (YLE 2023).

#### Land use

Forestry in the Sámi Homeland has been concentrated in the municipality of Inari, and there is a long history of forest conflicts between Metsähallitus (the Finnish Forest Administration) and reindeer herders (Jokinen 2014). The main conflicts were settled in 2009–2010, when Metsähallitus agreed with Sámi reindeer herders to keep important reindeer pasture areas free from logging (Jokinen 2014; Sarkki et al. 2022). Metsähallitus has also decided not to start mining or wind power projects in the Sámi Homeland until 2027. However, although the relations between reindeer herders and Metsähallitus have improved, some degree of controversy and tension remains between the private owners of forests and reindeer herders in the area (Sarkki et al. 2022).

Mineral prospecting is conducted in the Sámi Homeland, but it has not been developed into a large-scale mining industry. However, the mineral prospecting conducted earlier continues to cause negative affect and emotions in reindeer herders, including increasing concern of young herders for their future. Also, the rapid expansion of tourism into wilderness is problematic from the perspective of reindeer herding, because it increases uncontrollable traffic on pasture lands, disturbs reindeer and causes conflicts with reindeer herding (Turunen et al. 2016 unpubl.).

### Interviews

We conducted interviews in two herding cooperatives in the study area: Kaldoaivi (Gálddoaivi) in Utsjoki and Muotkatunturi (Muotkeduoddar) in Inari between January and December 2022 (Table 1). Our interview material consists of 11 conversational in-depth interviews, and 8 interviews conducted as part of a facilitated PPGIS survey. Four participants took part in both interviews, and thus, the total number of participants was 15. Themes of the interviews were the following: observations of environmental changes, extreme weather events and snow conditions; impacts of environmental changes, and extreme weather events on participants’ lives and their future perceptions regarding reindeer herding and Sámi culture; land use and land use-related conflicts. With the participants from Kaldoaivi, the impacts of Teno river fishing ban on their lives, fishing traditions and cultural continuity were also discussed.

**Table 1:** Characteristics of the two herding districts where interviews were conducted

	Kaldoaivi	Muotkatunturi
Surface area	2411 km^2^	2580 km^2^
No of reindeer owners	86	99
Highest allowed no of reindeer*	5300	6800
Protected areas	Kaldoaivi wilderness area	Lemmenjoki national park
		Muotkatunturi wilderness area
Common habitats	Mountain birch forests and heaths	Mountain birch forests and heaths
	Aapa and palsa mires	Aapa mires
	*Salix* scrubs	Pine forests in the southern part

^*^Reindeer Herders Association 2023

Twelve of the participants were male and three were female, aged between 19 and 65. Reindeer herding was the main occupation for 11 participants, while 4 had permanent jobs elsewhere, for example in education or communal services. Also, many of the participants with herding as the main occupation had part-time jobs, for example seasonal jobs in tourism. All participants had been involved in reindeer herding since childhood. They also actively practiced other traditional livelihoods for subsistence, such as cloudberry (*Rubus chamaemorus*) picking and fishing. For most participants from Kaldoaivi, Atlantic salmon fishing was a very important cultural and leisure activity and had provided subsistence during summer, but none of the participants earned a living from fishing. Some participants had practiced willow ptarmigan (*Lagopus lagopus*) trapping before, but had given up on it as willow ptarmigan populations had declined.

The interviews lasted between 30 min and two hours, with an average duration of one hour each. The interviews were conducted in person or via Teams by the authors (IM, VK, TR), audio recorded (with the exception of one case, where the participant did not want to have the interview recorded, and written notes were taken instead) and transcribed. The quotes from the interview transcripts presented in this article and accompanying table were translated by the authors from Finnish, the language of the interviews. The participants were informed about the aim of the study and about how the data will be used, and consent was obtained.

### PPGIS survey

The PPGIS survey was carried out using the Maptionnaire platform, a map-based online survey tool (new.maptionnaire.com). Information about the PPGIS survey and the survey link was distributed three times via email to the reindeer herders in Inari by the Reindeer Herders’ Association, two times in the local newspaper *Inarilainen* and a couple of times in social media (Facebook groups). The PPGIS survey included questions regarding land use conflicts and solutions, environmental change, appreciation of local environment and impacts of the COVID-19 pandemic. The relevance of the survey questions was tested by reindeer herders and other Sámi actors. Respondents were asked, for example, to mark on the maps areas where they have observed different kinds of environmental changes and areas where there are land use conflicts. Space for open-ended answers was provided. Respondents marked only a few areas with environmental changes on the map, and due to the low number of markings, we decided not to study the geographic locations marked by the respondents of the survey. However, in their open-ended answers, participants described the changes extensively and we thus use the open-ended answers to two questions of the survey as research material: 1. Have you observed changes in the environment of your herding district in a particular area or place during your lifetime? Please describe the change(s). What do you think is the reason for these changes? 2. What are the things in your community, reindeer herding district or in the environment of the district, that you appreciate the most, and you hope will still be there for future generations?

A total of 58 herders, most of them Sámi, participated in the survey. Of this total, 50 respondents completed the questionnaire online and 8 took part in the facilitated survey and interview. However, not all participants answered all questions. We used 35 open-ended answers to the above questions as material for this study, which includes the answers of the 8 interview participants.

### Analysis of interviews and PPGIS survey responses

The interviews and PPGIS survey responses were analyzed using qualitative thematic content analysis through which we aimed to gain an interpretative understanding of the material. First, we formulated three themes, which arose from our research aims: 1) Observed environmental changes; 2) Impacts of environmental changes on cultural continuity and well-being; 3) Expressions of ecological grief. We then examined the interview material to identify descriptions that represented these aspects. Second, we used deductive category application for the material regarding expressions of ecological grief and sought descriptions of each category. First, three pathways to ecological grief, defined by Cunsolo and Ellis (2018), were used as previously formulated, theoretically derived aspects of analysis (Table 2, categories 1–3). After analyzing the material, we identified a fourth category, which emerged from the interview material: grief associated with lack of decision-making power regarding natural resource governance, in particular decision-making related to species protection and land use. We then added this category to our category application. Our concept of environmental changes encompasses changes in weather, snow and ice conditions, alterations in landscapes and ecosystems as well as in species compositions, including decline in native species and alien species invasions.

**Table 2:** Categories of ecological grief used in this study. Categories 1–3 from Cunsolo and Ellis (2018), category 4 emerged from the interview material

Category	Description
1. Grief associated with physical ecological losses	Grief emerging from the physical disappearance of landscape and ecosystem degradation and decline/loss of species and attendant ways of life and culture
2. Grief associated with loss of environmental knowledge	Grief associated with loss of environmental knowledge. In indigenous people’s context, loss of their traditional ecological knowledge. In our analysis, we also included grief associated with loss of traditional practices in this category
3. Grief associated with anticipated future losses	Anticipatory grief for ecological changes that have not yet happened, including, in particular, grief over future losses of culture, livelihoods and ways of life
4. Grief associated with lack of power in environmental decision-making	Grief associated with the lack of power regarding natural resource governance, e.g., decision-making regarding species protection and land use planning

## Results

Participants identified numerous environmental changes, which have impacts on Sámi culture, and connections to well-being (Table 3). Ecological grief was expressed in connection to all major environmental concerns in the area: changes in winter weather and extreme weather events, Atlantic salmon decline and land use (Table 4), and was strongly associated with culture and ways of life which depend on nature.

**Table 3:** Observed environmental changes, cultural impacts and connections to well-being

Environmental change	Cultural/economic impact	Connection to well-being	Quotes
More frequent extreme winter weather events	Reindeer herding gets more difficult: there is increase in herders’ workload and costs when intensive winter supplementary feeding is needed	Stress due to increased workload and concerns over the future of reindeer herding as source of livelihood	“We had to take reindeer in winter enclosures for one month earlier than usually […] and keep them there for one month longer than normally. We needed to expand the enclosure and to buy tons of forage.” “Winter 2021–20 was the hardest in my life.” “There is no time to be with my family in wintertime.”
Unpredictability in weather, in particular in winter	Reindeer herding gets more difficult. Herders have to be prepared “for everything”	Stress due to uncertainty in weather and snow conditions and impacts on herding. Unpredictability causes concern over the future of herding livelihood	“Temperatures go up and down, it can be − 20 °C or − 30 °C today and around zero tomorrow. That has changed a lot. Like now, weather forecast for tomorrow is − 15 °C and day after tomorrow + 4 °C degrees.” “Snow conditions have changed so much. You no longer know what you get and when.”
Tree line shift to higher elevations and spread of pine	Changes in landscape and reindeer pastures. Open tundra is an important foraging habitat for reindeer and provides relief from insects during summer	No direct link to well-being. However, decline in populations of culturally important species and landscape transformations can lead to deterioration of cultural ties to the land (see: Jansson et al. 2015; Markkula et al. 2019)	“Open fell areas have been taken over by bushes.” “Pine has rooted into fell areas, tree line is ascending.”
Thawing of permafrost hummocks in palsa mires, and consequent decline in cloudberry	Palsa hummocks are an important element in the landscape Cloudberry is the most valuable natural product in the gathering tradition of the Sámi with high economic importance	Berry picking has many positive impacts on well-being: berries possess bioactive health properties, and berry picking is an important cultural activity that contributes to well-being of individuals and communities (see, e.g., Flint et al. 2011; Boulanger-Lapointe et al. 2019)	“Good cloudberry years are rare nowadays. There are no more cloudberries in places where they used to be, they used to grow on palsa hummocks.” “There are no more cloudberries as there used to be, the numbers have declined remarkably. This has a big impact. In the 1980s there were a lot of cloudberries, you could spend the whole summer in one bog, and you could never pick all of them.”

Environmental change	Cultural/economic impact	Connection to well-being	Quotes
Thinning of ice in lakes and rivers	Change in reindeer paths and movement, winter fishing gets more difficult	More accidents due to weak ice. Traditional knowledge (TK) regarding ice conditions is no longer valuable, which can evoke feelings of melancholy in particular among indigenous people, as TK is connected to both personal identity and to the whole cultural system of land-based knowledge	”It is no longer possible to predict ice conditions. It impacts movement of people, where you can go and which way to take. We have to go around those lakes, rivers and mires which are not frozen or are unpredictable.” “There is a small impact on herding because we have to wait longer [in autumn] to be able to cross rivers with reindeer.”
Moth outbreaks	Changes in landscape. Impacts on reindeer forage are first positive due to increase in grasses, but over time change to negative, as there is a decrease in mushrooms and loss of birch leaves, and pines take over the mountain birch forests. Moth outbreaks are also linked to willow ptarmigan decline	Landscape transformations can lead to deterioration of cultural ties to the land (see: Jansson et al. 2015) Can add to the cumulative negative impacts on reindeer herding and consequently to concerns over the future of the livelihood	“After moths have eaten birch, grasses and pine take over. Pines don’t grow very fast here but already in ten years they already grow quite a bit and after fifty years there are no longer open fells but forests [in those areas where moth outbreaks occurred].”
Willow ptarmigan decline	Willow ptarmigan is an important game species in Sámi cultural tradition TK related to ptarmigan trapping may diminish. Parts of ptarmigan are also used in traditional handicrafts	Decline in culturally important species can lead to deterioration of cultural ties to the land (see: Jansson et al. 2015; Markkula et al. 2019). Loss of traditions and TK can evoke feelings of melancholy	“There’s been great decline. In my childhood ptarmigan was very abundant, and there were many people who were professional ptarmigan trappers. Now it is no use to trap [ptarmigan], they are so few.”
Atlantic salmon decline	Atlantic salmon is a cultural keystone species in the area Population decline has caused big economic losses and concerns over the continuity of fishing culture	Stress, anxiety and fear over cultural loss caused by the decline of cultural keystone species and by the fishing ban that followed	“There is something big happening in the [Barents] sea, maybe there is no more prey for Atlantic salmon, or the prey species have moved to somewhere else […] This might have happened because sea water temperatures have risen.”
Pink salmon invasion	River eutrophication, decline in native species and water quality. Rotting salmon on river shores may also bring new/more predators to the area and can impact aesthetic landscape values	Fear that pink salmon will eventually override Atlantic salmon in the River Teno. Possible impacts due to eutrophication/poor water quality	“Pink salmon is so abundant now. Last summer the river shores were full of rotten salmon.”

**Table 4:** Expressions of ecological grief in relation to changing winter weather, extreme weather events, Atlantic salmon decline and other environmental changes

	Change in winter weather and extreme weather events	Atlantic salmon decline	Land use changes
1. Grief associated with ecological losses	Winter is no longer winter	It was hard mentally. It [Atlantic salmon decline and the following fishing ban] made me feel as if I had lost an arm, because it [fishing] had been with me since childhood It [Atlantic salmon decline and following fishing ban] causes psychological distress	The threat [posed by land use] affects your mental well-being and your ability to cope. What I meant that you constantly need to defend your livelihood
2. Grief associated with loss of environmental knowledge and cultural practices	You can no longer rely on old ways of forecasting the weather Our traditional knowledge about weather, reindeer movement, pasture use, it is no longer useful It has changed so that you no longer know what comes and when [referring to snow and winter weather]	Kulkutus [traditional fishing practice] has almost disappeared already. They [authorities] managed to stop it when they passed the time limit. […] I feel melancholic [about the loss of fishing traditions] It [the fishing ban] is a cultural shut-down	But yes, some knowledge regarding gathering, moving and herding [reindeer] has already disappeared. Knowledge regarding natural pasture rotation and the like. We have to invent new knowledge all the time and, at the moment, the old [knowledge] is not seen as meaningful. It would be useful to have traditional knowledge, and even more in the future, but this is where we are at now
3. Grief associated with anticipated future losses	I am very concerned, afraid of what’s going to happen. Is it possible to continue [herding] this way. Maybe I still can but what about the next generations. Will there be prerequisites for it [herding]? Will reindeer be able to live here, or anything or anyone.* I’ve been thinking about it a lot, whether reindeer herding will even exist in 20 years. It is hard to say. It is possible that climate change will ruin it all, if bad winters become frequent, or someone just decides to prohibit reindeer herding, or they make herding very difficult. On the other hand, I do not see any obstacles, reasons why young people could not become herders. Well, there is climate change, and reindeer herding is a quite sensitive livelihood…I mean, they suddenly prohibited fishing too. Many people here earned their living from fishing, and it just stopped, in a flash	Future [of Sámi fishing culture] seems very uncertain. I am afraid that the traditional fishing practices will disappear, soon there will be no one left to practice them It is not possible to maintain salmon fishing culture anymore, it will slowly disappear	Land use [for purposes other than reindeer herding] has a big impact on your sense of safety, which is a basic need. It impacts your whole existence. For example plans for the Arctic railway, or wind farms, or mines can create an existential crisis
4. Grief associated with lack of power in environmental decision-making	Right now it's really good, but I am afraid, because you never know what will happen in the nature in the first place, and what will happen with the authorities. In the evening, when I go to sleep, I often think about the future, whether reindeer herding still exists 20 years from now. […] I don’t know, at the moment reindeer herding is profitable here. But in 20 years, who knows if it is even allowed to have reindeer anymore.*	I think the fishing restrictions are unequal. I am concerned about the elderly people in particular. If you think of an old lady, she owns 1 km of the River Teno shoreline and has lived all her live by the river. Her parents and grandparents lived here too, and they all ate Atlantic salmon. She has never practiced overfishing, she just took what she needed for food. And now she has no rights at all, she is not allowed to catch even one salmon to get the taste of it It [fishing ban] is part of the injustice against Sámi people that is evident everywhere. […] The rights of Sámi to practice their culture [set in Constitution of Finland] are not realized	Sámi culture will stay alive, but it will change. Cultures always change, this is how it goes. But I am afraid Sámi culture will be nothing more than an ornament in the future. Even people of my age, some of them think: what difference does it make…being Sámi, you know, if you think about the River Teno agreement and the fishing ban for example, the Sámi rights did not count at all

*These two quotes are also included in another article, Markkula et al. 2024. "Changing winter landscapes: Extreme weather events and meanings of snow for Sámi reindeer herders", in press

Based on the results, the study area has undergone multiple environmental changes in recent years and decades, and their impacts on reindeer herding are many: moth outbreaks, tree line shifts and the associated spread of pine change pasture qualities; extreme weather events prevent reindeer from digging for food, and thinning of ice in lakes and rivers changes reindeer movement and migration routes. Under extreme weather conditions, such as unusually thick snow covers, repeated freeze–thaw cycles and ground ice, extensive supplementary feeding of reindeer is needed, which increases the workload or reindeer herders and the costs of herding. Combined with losses of reindeer in extreme winters, the economic impact is significant.

Moreover, unpredictability in weather causes both acute and long-term stress. When it is not possible to predict the weather conditions, herders feel that they need to be “prepared for everything”. At the same time, there is a growing concern over the future of reindeer herding as a livelihood. When interviews took place, the participants had just experienced two winters which were very difficult for reindeer herding: many participants described the winter 2019–2020 as the snowiest they had ever seen and the winter 2021–2022 as the most difficult in their whole lifetime. These extreme winter weather events, together with more gradual changes in winter weather, evoked grief over loss of traditional knowledge and grief associated with anticipated future losses among the participants (categories 2 and 3, Table 2). Winters 2019–20 and 2021–22 were seen as harbinger of future by the participants and evoked fear over the future of reindeer herding as a source of livelihood. Most of them had been wondering whether reindeer herding still exists 20 years from now. Some of the participants expressed concerns that consideration of combined impacts of climate change, land use and economic issues may make reindeer herding as a livelihood appear as an impossible or undesirable way of life for younger generations.

Climate change and land use were seen as the greatest threats to the continuity of the reindeer herding livelihood. One of the participants described how land use, e.g., forestry, plans for a railway, windfarms or mine construction “impact your whole existence” and “can create an existential crisis” (Table 4). Participants emphasized that climate change and land use can have significant combined effects on reindeer herding. One example is the combined effect of logging and extreme winter weather events. Epiphytic lichen growing in old-growth forests are important source of winter forage for reindeer, particularly in winters when deep and hard snow or ground—ice conditions prevent reindeer from digging for food. Logging in old-growth forests leads to a decrease in winter forage for reindeer and makes adaptation to extreme winter weather events even more difficult. Results also indicate that even test drilling for ore and land use plans which are still at the planning phase have a negative impact on well-being as they increase the “load of worries”.

Land use and Atlantic salmon decline evoked grief associated with physical ecological losses, losses of traditional knowledge and practices and anticipated future losses among the participants (Categories 1–3, Table 4). Participants expressed sadness, deep emotional attachment to the Atlantic salmon and feelings of inequality in the face of the fishing restrictions. Atlantic salmon population decline and the following fishing ban, which came into force in summer 2021, were strongly reflected in the expressions of ecological grief among Kaldoaivi herders. Participants reported melancholy and physical distress. Grief over loss of cultural practices anticipated future losses were evident: *I am afraid that the traditional fishing practices will disappear, soon there will be no one left to practice them* (Table 4). The Atlantic salmon fishing ban also caused anticipatory grief connected to the future of reindeer herding as a livelihood, which is well illustrated by this quote from a young herder: *I’ve been thinking about it a lot, whether reindeer herding will even exist in 20 years. It is hard to say. It is possible that climate change will ruin it all, if bad winters become frequent, or someone just decides to prohibit reindeer herding, or they make herding very difficult. […]I mean, they suddenly prohibited fishing too* (see the full quote in Table 4). This quote embodies the interconnectedness of everything: the traditional livelihoods are all part of local life and culture, bound to each other and to the current social and political environment.

Given the high number of answers expressing anxiety caused by the fishing ban and land use decisions, we added a fourth ecological grief category to our study: grief associated with lack of decision-making power regarding natural resource governance (Category 4; Tables 2, 4). Regarding the fishing ban, participants found it necessary to regulate Atlantic salmon fishing on River Teno, as they had witnessed the population decline themselves. However, at the same time, they felt that the total fishing ban was just another political decision that would weaken the rights of the Sámi as an Indigenous people: *It [fishing ban] is part of the injustice against Sámi people that is evident everywhere.* The fishing ban was construed as part of a historical continuum of resource use and fishing politics concerning River Teno.

## Discussion

### Climate change, cultural continuity and well-being

Participants, who were either full- or part-time reindeer herders, considered herding as an essential carrier of Sámi culture, and many of them mentioned that keeping culture and traditions alive is one of the main motives for being a herder. At the same time, they estimated that due to combined effects of climate change and competing land uses, there will be less herders in the future, and passing traditions on to future generations will become increasingly difficult. It also becomes harder to continue with the herding culture, when the traditional knowledge is no longer valid, and the traditional grazing areas and routes to transfer reindeer cannot be used anymore.

Questions of cultural continuity are thus related to extreme winter weather events, which are getting more frequent in the warming climate. At the same time, other traditional livelihoods which contribute to cultural continuity are affected by climate change. Fishing, willow ptarmigan trapping and cloudberry picking have long historical roots and can maintain cultural ties to the land (e.g., Jansson et al. 2015; Markkula et al. 2019). The first records of Atlantic salmon fishing in the Teno river valley date back to the sixteenth century, and fishing is an important cultural activity, which carries on specific fishing terminology and traditional knowledge regarding weather, water levels, river ecosystem, fishing practices and beliefs (Sámi Parliament 2021). Against this historical backdrop, the Atlantic salmon decline and the River Teno fishing ban represent significant examples of how climate change impacts cultural continuity.

Previous studies have demonstrated that cultural continuity is connected to health and wellness benefits, in particular in Indigenous communities, and is associated with strong cultural identity, positive identity and self-esteem (e.g., Wexler 2014; Auger 2016; Kowalczewski and Klein 2018). Cultural continuity can be defined as “intergenerational cultural connectedness, which is maintained through intact families and the engagement of elders, who pass traditions to subsequent generations” (Loppie Reading and Wien 2009). Cultural connectedness, on which cultural continuity builds, has been defined as “the extent to which youth is integrated within his or her native [or Indigenous] culture” (Snowshoe et al. 2014). The current situation of the River Teno is a good example of disruption of cultural continuity: the Atlantic salmon fishing culture on the river is no longer dynamic, and it is very hard, if not impossible, to pass traditions on to younger generations. These issues were reflected in the interviews: participants expressed concern in particular over the well-being of elderly people, for whom traditional fishing practices are a key element in life. The fishing ban was described as a cultural shut-down and the Atlantic salmon fishing culture in the area was expected to slowly disappear. This is in line with a recent report regarding the social impacts of the River Teno fishing ban, in which Utsjoki inhabitants, both Sámi and Finnish, expressed fear and melancholy over the loss of salmon fishing culture (Abernethy et al. 2022).

Nature itself is an important source of well-being, and time spent in nature is beneficial for mental health (Timlin et al. 2021). For example, berries are known to be good for health due to their bioactive properties, but also because berry picking is a social and cultural outdoor activity which makes people happy (Flint et al. 2011; Boulanger-Lapointe et al. 2019; see Table 3). Moreover, it is common that people who live in close connection to the surrounding nature invest themselves emotionally with plants and animals and have cultural, emotional and mental relations with the land they live on (e.g., Sakakibara 2020). This kind of emotional investment was evident also in our interviews. Participants described their connection with reindeer, the happiness they feel when in the woods and when autumn round-ups start, and their joy of seeing the new-born calves in spring. Some of them also expressed worry over how reindeer and other animals will survive in a warmer world. Participants also expressed deep emotional attachment to the Atlantic salmon, and thus, a fishing ban can be expected to affect their mental health in at least two ways: first, by the lack of positive well-being impacts of fishing, time spent on the river, and being in contact with nature, and second, through feelings of melancholy and grief over the loss of culture and traditional practices. Moreover, Atlantic salmon fishing is an essential constituent of individual and social well-being, and fishing on the River Teno is an integrative element in the local community (Hiedanpää et al. 2020; Abernethy et al. 2022). This study, in line with earlier studies, indicates that environmental changes are often perceived and experienced through subsistence practices, and environmental change, cultural continuity and local economy are tightly connected (e.g., Moerlein and Carothers 2012; Ready and Collins 2021). Human well-being is often a result of interactions between these factors.

### Environmental change and ecological grief

Studying ecological grief can enhance our understanding of how climate and environmental changes affect people’s well-being and help to define environmental losses that matter to local people (Ojala et al. 2021). In this study, we applied the categorization of the pathways of ecological grief by Cunsolo and Ellis (2018). These categories, even though partly overlapping, can help to understand whether grief is a response to acute or gradual losses (grief associated with physical ecological losses), anticipated future losses and/or connected to cultural continuity (loss of environmental knowledge and cultural practices).

Grief associated with physical losses typically emerges after extreme weather events or natural disasters, but can also emerge in response to slow, gradual and ongoing ecological changes, such as longer-term changes in weather patterns, landscapes or ecosystems (Cunsolo and Ellis 2018). Extreme winter weather events did not evoke strong expressions of this kind of grief in our study. This could be because herders have long experience of dealing with difficult and unpredictable winter weather and are skilled and well prepared to cope with exceptional conditions (Vuojala-Magga et al. 2011; Turunen et al. 2016). However, many of these strategies are not always welcomed but forced, and adaptation options are limited (e.g., Rasmus et al. 2023). This is reflected in expressions of grief over loss of traditional knowledge and anticipated future losses in relation to extreme weather events and climate change in general (Categories 2 and 3, Table 2). The pressure to adapt to changing climate is strong, and it is clear that adaptation actions, such as supplementary winter feeding and keeping reindeer in enclosures, will finally lead to long-lasting or permanent alterations in reindeer herding practices and culture (e.g., Rasmus et al. 2023). Even though cultures are dynamic and will always inevitably change, sudden and forced changes often require mourning.

Stress and grief felt by reindeer herders over the unpredictability in weather resonates with previous research. In arid areas in Ghana and Australia, farmers have lost confidence in their ability to predict future weather and have started to question their environmental wisdom (Ellis and Albrecht 2017; Amoak et al. 2023). In Nunatsiavut in Canada, Inuit are increasingly questioning their identity when they cannot go out on the land because ice conditions are too unpredictable, leading to a situation where it is not possible to know “what you’re good at […] what your self-worth is, not knowing what you should be doing with your time” (Cunsolo et al. 2012).

Atlantic salmon decline and fishing ban evoked strong expressions of grief over the loss of species, traditional knowledge and culture and anticipated future losses. While Atlantic salmon decline, which has accelerated over recent years, represents a gradual change, the fishing ban that followed created a new, acute situation, which is reflected in participant’s responses. The significance of Atlantic salmon to the identity of the Sámi and other local people clearly emerges in the participants’ descriptions of grief. Their descriptions reflect the role of the Atlantic salmon as part of individual participants’ life histories, of which the following quote is particularly illustrative: *It [Atlantic salmon decline and the following fishing ban] made me feel as if I had lost an arm, because it [fishing] had been with me since childhood* (see Table 4). Similar emotions have been reported by Cunsolo et al. (2020) among Inuit in Canada where Government of Newfoundland and Labrador issued a total hunting ban on caribou in 2013 after dramatic population decline. Inuit in Labrador adapted to the loss of access to caribou by substituting caribou for moose and by continuing to hunt for other species. This adaptation, however, had an emotional cost in the form of grief, as hunters felt that there is “nothing that could replace the caribou”. They saw that the hunting ban was going to lead to a total change of culture and degradation of traditional knowledge. Like the Sámi living by the River Teno, they felt that an important part of their identity was taken away and expressed special concern for elders, who experience deep sadness over the cultural discontinuity (Cunsolo et al. 2020). Also, a recent study conducted among farmers in Ghana reported that loss of environmental knowledge associated with climate change and endangered species was a significant source of ecological grief, and elderly people were fearing that their knowledge might just end within their generation (Amoak et al. 2023).

The melancholic feeling that the River Teno is no longer the river it used to be was strongly present in the interviews: There are no more rowing boats on the river, invasive pink salmon has increased enormously, and masses of their post-spawn carcasses are decaying on the riverbanks and in the water, contributing to eutrophication of the river. This resonates with the concept of “solastalgia” by Glenn Albrecht (2005; 2017), meaning homesickness that you feel while you are still at home. Feelings of solastalgia were also connected to the changes in winter weather, as many participants described how winters are not what they use to be. These descriptions of weather and snow changes resonate with the opening words of Sheila Watt-Cloutier to her book *Right to be cold* (2018): “The world I was born into has changed forever”.

Previously, feelings of solastalgia have been documented in different parts of the world, e.g., among Inuit communities in Labrador after caribou hunting ban was established (Cunsolo et al. 2020), in relation to changes in soundscapes due to decline and change in bird populations (Krause 2017) and after population collapse of sparrows in London (Whale and Ginn 2017). While the concepts of solastalgia and ecological grief are partly overlapping, the concept of solastalgia emphasizes the idea that grief people feel for ecological losses is often place specific and connected to the meanings of home.

### Ecological grief and environmental decision-making

Our results suggest that ecological grief is not only associated with ecological losses, but also with political decisions regarding natural resource governance. In the interviews, participants expressed grief, which did not entirely fit in any of the three deductive categories. This kind of ecological grief associated with lack of power in environmental decision-making can emerge in situations where local or Indigenous Peoples’ rights for harvesting are restricted by political decisions. The River Teno fishing ban is a good example of this. Also, as was noted before, all extensive land use, such as forestry and windfarm, railway or mine constructions, causes strong emotional responses, including anxiety, grief and anger, which arise both from the environmental loss and from the minor or non-existing possibilities to impact decision-making processes.

Recognizing ecological grief and solastalgia as part of land use planning processes is important for at least two reasons: to ensure meaningful participation to prevent grief which arises from decision-making itself, and to acknowledge place and Indigenous and local people’s relationships with it. According to Howard (2020), “planning for ecological grief, with place at the forefront, is a strong strategy for creating locally-sensitive programming and policies, enhancing resilience, and decreasing grief-related burdens”. Grief is a statement of value (Howard 2020), which makes it an important aspect to consider in land use planning and decision-making.

Howard writes that biodiversity loss places earth-based value systems, subsistence lifestyles and cultural practices at a high risk of future loss, and that these factors often connect to histories of marginalization and systematic de-realization of Indigenous concerns, aggravating experiences of grief (Howard 2020). Addressing environment as kinship, Braun (2017) describes how Lakota people mourn the death of a buffalo as an individual and a species, but also as a symbol of historical trauma. Histories of marginalization were also reflected in our interviews. One participant described the impacts of the fishing ban on Sámi identity and discussed the contradictions between fishing politics and rights of Sámi to practice their culture. By asking *what difference does it make—being a Sámi* (see Table 4), he referred to the history of marginalization, as well as to the 2017 Fishing Agreement and the current situation in River Teno, claiming that Sámi rights to practice their culture do not actualize.

Some of the participants in our study pointed out that they may be able to adapt to the changing situation, to fish other species instead, e.g., pike or white fish, or start to use pink salmon, or expressed hope that Atlantic salmon populations will recover in the near future, and fishing ban is no longer needed. However, despite some adaptation possibilities, many felt that the situation is beyond their control. Also Abernethy et al. (2022) have documented how Sámi in Utsjoki experienced loss of control over their lives and self-determination as a result of the Atlantic salmon fishing ban. Similar feelings were expressed by the participants of our study in relation to land use decisions they could not influence and have been reported in previous studies on reindeer herding (e.g., Löf 2013; Furberg et al. 2013). Previously, Cunsolo et al. (2012) have documented how Inuit living in Labrador experienced grief over being unable to protect the land from the impacts of climate change, feeling helpless while bearing witness to the changes they could not stop from happening: “you’re in no place to control that yourself”. Regarding extreme weather events, participants expressed wishes for more support from the government during difficult times. They were hoping that Finland would adopt practices that have been applied in Norway, where supplementary hay for reindeer is bought and transported to reindeer herding areas by the state to help reindeer and herders to survive extreme weather events. By this, they brought up that land use decisions, local and national-level politics and practices can thus create either hope or hopelessness among local and Indigenous People. Finally, it should be noted that reindeer herders who participated in our study had different adaptation strategies, such as flexibility in subsistence and economic diversification, and strong resilience and coping capacity in particular in relation to extreme winter weather events. Previous studies have noted that Indigenous Peoples and local communities have, throughout their history, fostered flexible subsistence to deal with climate variability and should not be presented so much as victims of climate change but as resilient actors (Moerlein and Carothers 2012; Fawcett et al. 2018; Eerkes-Medrano and Huntington 2021). However, also ecological mourning can be an adaptive process, because in order to accept losses, it is necessary to go through grief (Ojala et al. 2021). Previous research has shown that nonclinical worry and grief can be constructive and lead to adaptive behavior. However, to be able to act, people need to perceive their situation as controllable at least to some extent (Tallis et al. 1994; Gross 2016; see also Ojala et al. 2021). Thus, when it comes to local decision-making and land use planning processes, questions of grief and control and their relations to meaningful participation are important issues to consider. Meaningful participation should include bearing witness to the grief of others and listening to their speak-outs and storytelling in order to understand experiences of ecological grief that are often left unnoticed (see also Howard 2020).

## Conclusions

We studied cultural impacts of climate change and expressions of ecological grief among members of reindeer herding communities in the Sámi Homeland in Finland. While climate change emerged as one of the main concerns among herders, land use and natural resource governance are equally important for the continuity of their traditional livelihoods, having important combined effects on cultural continuity. Moreover, climate change can force political decisions and adaptation practices, which create stress, concern and ecological grief among Indigenous and local people whose lives and livelihoods are closely connected to and depend on the surrounding nature.

Ecological grief was expressed in connection to all major environmental concerns in the area, and it seems clear that climate and environmental change in the Sámi Homeland challenge local and Indigenous people at deep personal and cultural levels. Ecological grief is both a local phenomenon and a global phenomenon in that it is often connected to local losses of place, species, ways of life and traditional or local knowledge, but similar expressions of grief and concern over the loss of nature, knowledge and cultural practices are found in different parts of the world. Expressions of ecological grief are often tightly connected to local-level impacts of climate change. Thus, addressing ecological grief can add to the research investigating climate change impacts, in particular to studies attempting to create cross-cultural observation systems (e.g., Reyes-Garcia et al. 2019).

Our results suggest that ecological grief is not only associated with ecological losses, but also with political decisions regarding natural resource governance and the inability to genuinely influence them. It is our hope that this finding will shed light on the importance of meaningful participation of Indigenous Peoples in decision-making processes. Addressing the concept of ecological grief can help to include ecological places, values and identities—which are often intangible, hard to measure or invisible—into decision-making and land use planning.

## References

[CR1] Abernethy, P., Saijets, J., Jokinen, M., Knuuttila, M. and Hiedanpää, J. 2022. Societal impacts of Teno river salmon fishing ban (in Finnish). Luonnonvarakeskus 2022.

[CR2] Albrecht G (2005). “Solastalgia: A new concept in human health and identity. PAN (philosophy, Activism, Nature).

[CR3] Albrecht G, Nature Mourning (2017). Solastalgia and the new mourning. Cunsolo and Landman.

[CR4] AMAP (2017). Adaptation actions for a changing arctic perspectives from the barents area.

[CR5] Amoak D, Kwao B, Ishola TO, Mohammed K (2023). Climate change induced ecological grief among smallholder farmers in semi-arid Ghana. SN Social Sciences.

[CR6] Auger, M. 2016. Cultural continuity as a determinant of indigenous peoples’ health: A metasynthesis of qualitative research in Canada and the United States. *The International Indigenous Policy Journal* 7.

[CR7] Bailey H, Hubbard A, Klein ES, Mustonen K, Akers PD, Mattila H, Welker JM (2021). Arctic sea ice loss fuels European extreme snowfall. Nature Geoscience.

[CR8] Boulanger-Lapointe, N., J. Gérin-Lajoie, L. Siegwart Collier, S. Desrosiers, C. Spiech, G.H.R. Henry, L. Hermanutz, E. Lévesque, et al. 2019. Berry plants and berry picking in inuit nunangat: Traditions in a changing socio-ecological landscape. *Human Ecology* 47: 81–93.

[CR9] Braun S, Nature Mourning (2017). Mourning ourselves and/as our relatives: environment as kinship. Cunsolo and Landman.

[CR10] Buchwal A, Sullivan PF, Macias-Fauria M, Post E, Myers-Smith I, Stroeve JC, Blok D, Tape KD (2020). Divergence of Arctic shrub growth associated with sea ice decline. Proceedings of the National Academy of Sciences.

[CR11] Cunsolo AW (2012). Climate change as the work of mourning. Ethics and the Environment.

[CR12] Cunsolo A, Ellis NR (2018). Ecological grief as a mental health response to climate change-related loss. Nature Climate Change.

[CR13] Cunsolo A, Landman K, Nature Mourning (2017). Introduction: To mourn beyond the human. Cunsolo and Landman.

[CR14] Cunsolo Stephenson AWE, Allen J, Bourque F, Drossos A, Elgarøy S, Kral MJ, Mauro I (2015). Examining relationships between climate change and mental health in the circumpolar north. Regional Environmental Change.

[CR15] Cunsolo, A., D. Borish, S-L. Harper, J. Snook, I. Shiwak, M. Wood and The Herd Caribou Project Steering Committee. 2020. “You can never replace the caribou”: Inuit experiences of ecological grief from caribou declines. *American Imago* 77: 31–59.

[CR16] Cunsolo Willox, A. S.-L. Harper, J.D. Ford, V.L. Edge, K. Landman, K. Houle, S. Blake and C. Wolfrey. 2013. Climate change and mental health: An exploratory case study from rigolet, nunatsiavut, Canada. *Climatic Change* 121: 255–270.

[CR17] Cunsolo Willox, A., S.-L. Harper, J.D. Ford, K. Landman, K. Houle, V.L. Edge and the Rigolet Inuit Community Government. 2012. From this place and of this place: Climate change, sense of place, and health in Nunatsiavut, Canada. *Social Science and Medicine* 75: 538–547. 10.1016/j.socscimed.2012.03.043 22595069

[CR18] Czorlich Y, Aykanat T, Erkinaro J, Orell P, Primmer CR (2022). Rapid evolution in salmon life history induced by direct and indirect effects of fishing. Science.

[CR19] Dadswell M, Spares A, Reader J, McLean M, McDermott T, Samways K, Lilly J (2022). The decline and impending collapse of the Atlantic salmon (*Salmo salar*) population in the North Atlantic Ocean: A review of possible causes. Reviews in Fisheries Science and Aquaculture.

[CR20] Dodd W, Scott P, Howard C, Scott C, Rose C, Cunsolo A, Orbinski J (2018). Lived experience of a record wildfire season in the Northwest territories, Canada. Canadian Journal of Public Health.

[CR21] Durkalec A, Furgal C, Skinner MW, Sheldon T (2015). Climate change influences on environment as a determinant of indigenous health: Relationships to place, sea ice, and health in an Inuit community. Social Science and Medicine.

[CR22] Eerkes-Medrano L, Huntington HP (2021). Untold stories: Indigenous knowledge beyond the changing arctic cryosphere. Frontiers in Climate.

[CR23] Ellis NR, Albrecht G (2017). Climate change threats to family farmers' sense of place and mental wellbeing: A case study from the Western Australian wheatbelt. Social Science & Medicine.

[CR24] Fawcett D, Pearce T, Notaina R, Ford J, Collings P (2018). Inuit adaptability to changing environmental conditions over an 11-year period in Ulukhaktok, Northwest Territories. Polar Record.

[CR26] Flint CG, Robinson ES, Kellogg J, Ferguson G, BouFajreldin L, Dolan M, Raskin I, Lila MA (2011). Promoting Wellness in Alaskan villages: Integrating traditional knowledge and science of wild berries. EcoHealth.

[CR27] Furberg M, Evengård B, Nilsson M (2013). Facing the limit of resilience: Perceptions of climate change among reindeer herding Sami in Sweden. International Journal of Circumpolar Health.

[CR28] Gross R (2016). Understanding grief: An introduction.

[CR29] Hiedanpää J, Saijets J, Jounela P, Jokinen M, Sarkki S (2020). Beliefs in conflict: The management of Teno Atlantic salmon in the Sámi Homeland in Finland. Environmental Management.

[CR30] Holmberg, A. 2018. Bivdit Luosa – To ask for salmon. Saami traditional knowledge on salmon and the river Deatnu: In research and decision-making. Master thesis, The Arctic University of Norway.

[CR31] Howard, Q. 2020. Navigating loss and damage: Making space for ecological grief in the climate adaptation planning process. Doctoral thesis, The University of Guelph.

[CR32] Jaakkola JJK, Juntunen S, Näkkäläjärvi K (2018). The holistic effects of climate change on the culture, well-being, and health of the Saami, the only Indigenous people in the European Union. Current Environmental Health Reports.

[CR33] Jansson R, Nilsson C, Keskitalo ECH, Vlasova T, Sutinen ML, Moen J, Chapin FS (2015). Future changes in the supply of goods and services from natural ecosystems: Prospects for the European north. Ecololgy and Society.

[CR34] Jokinen M, Katila In (2014). Heated and frozen forest conflicts: Cultural sustainability and forest management in arctic Finland. Forests under pressure: Local responses to global issues.

[CR35] Joona T (2020). ILO convention No. 169 and the governance of indigenous identity in Finland: Recent developments. The International Journal of Human Rights.

[CR36] Knuuttila, M., Lankia, T. Länsman, M., Pouta, E., Vatanen, E., and Venesjärvi, R. 2020. Regional economic effects of Altantic salmon and the development of fishing tourism (in Finnish). Luonnonvara- ja biotalouden tutkimus 85/2020.

[CR37] Kowalczewski E, Klein J (2018). Sámi youth health, the role of climate change, and unique health-seeking behaviour. International Journal of Circumpolar Health.

[CR38] Krause B, Nature Mourning (2017). Mourning the loss of wild soundscapes: a rationale for content when experiencing natural sound. Cunsolo and Landman.

[CR39] Kumpula, J., Jokinen, M., Siitari, J. and Siitari, S. 2020. Weather, snow and nature conditions during winter 2019–2020 and impacts on reindeer herding (in Finnish). Luonnonvara- ja biotalouden tutkimus 58/2020.

[CR40] Kumpula, J., Rämö, S., Siitari, J., Holkeri, L., Pekkarinen, A-J. and Tauriainen, J. 2022. Snow conditions during winter 2021–2022 and impacts on reindeer herding (in Finnish). Luonnonvara- ja biotalouden tutkimus 71/2022.

[CR41] Landauer M, Rasmus S, Forbes BC (2021). What drives reindeer management in Finland towards social and ecological tipping points?. Regional Environmental Change.

[CR42] Löf A (2013). Examining limits and barriers to climate change adaptation in an Indigenous reindeer herding community. Climate and Development.

[CR43] Luomaranta A, Aalto J, Jylhä K (2019). Snow cover trends in Finland over 1961–2014 based on gridded snow depth observations. International Journal of Climatology.

[CR44] Markkula I, Turunen M, Rasmus S (2019). A review of climate change impacts on the ecosystem services in the Saami Homeland in Finland. Science of the Total Environment.

[CR45] Middleton J, Cunsolo A, Pollock N, Jones-Bitton A, Wood M, Shiwak I, Flowers C, Harper S (2021). Temperature and place associations with Inuit mental health in the context of climate change. Environmental Research.

[CR46] Moerlein KJ, Carothers C (2012). Total environment of change: impacts of climate change and social transitions on subsistence fisheries in northwest Alaska. Ecology and Society.

[CR47] Ojala M, Cunsolo A, Ogunbode C, Middleton J (2021). Anxiety, worry, and grief in a time of environmental and climate crisis: A narrative review. Annual Review of Environment and Resources.

[CR48] Sámi Parliament. 2021. Statement of the Sámi parliament on the government decree on the temporary prohibition of salmon fishing in the teno river basin (in Finnish).

[CR49] Rantanen M, Karpechko AY, Lipponen A, Nordling K, Hyvärinen O, Ruosteenoja K, Vihma T, Laaksonen A (2022). The Arctic has warmed nearly four times faster than the globe since 1979. Communications Earth & Environment.

[CR50] Rasmus S, Turunen M, Luomaranta A, Kivinen S, Jylhä K, Räihä J (2020). Climate change and reindeer management in Finland: Co-analysis of practitioner knowledge and meteorological data for better adaptation. Science of the Total Environment.

[CR51] Rasmus, S., Landauer, M., Lehtonen, I., Mettiäinen, I., Sorvali, J., Kumpula, J., Tuomenvirta, H., and Turunen, M. 2023. Adaptation of reindeer herding to climate change, Final report of Climini-project (in Finnish, English summary). Arktisen keskuksen tiedotteita 64.

[CR52] Reading CL, Wien F (2009). Health inequalities and social determinants of Aboriginal Peoples’ health.

[CR53] Ready E, Collins P (2021). All the problems in the community are multifaceted and related to each other: Inuit concerns in an era of climate change. American Journal of Human Biology.

[CR54] Reyes-Garcia V, Garcia-del-Amo D, Benyei P, Fernández-Llamazares A, Gravani K, Junqueira AB, Labeyrie V, Li X (2019). A collaborative approach to bring insights from local observations of climate change impacts into global climate change research. Current Opinion in Environmental Sustainability.

[CR55] Richert J, Leffler J, Spalinger D, Welker JM (2021). Snowier winters extend autumn availability of high-quality forage for caribou in Arctic Alaska. Ecosphere.

[CR56] Sakakibara C (2020). Whale Snow: Iñupiat, climate change, and multispecies resilience in Arctic Alaska.

[CR57] Sarkki S, Jokinen M, Heikkinen HI, Nijnik N, Melnykovuch M, Kluvánková T (2022). “Going out to get in”—Roles of forest conflicts in bottom-linked environmental governance progressing toward socio-political innovations. Environmental Policy and Governance.

[CR58] Snowshoe A, Crooks CV, Tremblay PF, Craig WM, Hinson RE (2014). Development of a cultural connectedness scale for first nations youth. American Psychological Association.

[CR59] Tallis F, Davey GCL, Capuzzo N, Davey GCL, Tallis F (1994). The phenomenology of non-pathological worry: a preliminary investigation. Worrying perspectives on theory, assessment and treatment.

[CR60] Teno Fishing Act 2017 176/2017. https://www.finlex.fi/fi/laki/alkup/2017/20170176.

[CR25] The Constitution of Finland §17/1999. https://www.finlex.fi/fi/laki/kaannokset/1999/en19990731.pdf.

[CR61] Timlin U, Ingimundarson JH, Jungsberg L, Kauppila S, Larsen JN, Nordstrom T, Scheerf J, Schweitzer P (2021). Living conditions and mental wellness in a changing climate and environment: Focus on community voices and perceived environmental and adaptation factors in Greenland. Heliyon.

[CR62] Turunen MT, Rasmus S, Bavay M, Ruosteenoja K, Heiskanen J (2016). Coping with difficult weather and snow conditions: Reindeer herders' views on climate change impacts and coping strategies. Climate Risk Management.

[CR63] Vuojala-Magga T, Turunen M, Ryyppö T, Tennberg M (2011). Resonance strategies of Sami reindeer herding during climatically extreme years in northernmost Finland in 1970–2007. Arctic.

[CR64] Watt-Cloutier S (2018). The right to be cold: One woman’s fight to protect the arctic and save the planet from climate change.

[CR65] Wexler L (2014). Looking across three generations of Alaska Natives to explore how culture fosters Indigenous resilience. Transcultural Psychiatry.

[CR66] Whale H, Ginn F, Nature Mourning (2017). In the absense of sparrows. Cunsolo and Landman.

[CR67] YLE. 2023. Finnish broadcasting company, News 16.8.2023, https://yle.fi/a/74-20045450.

